# Midkine-a Regulates the Formation of a Fibrotic Scar During Zebrafish Heart Regeneration

**DOI:** 10.3389/fcell.2021.669439

**Published:** 2021-05-07

**Authors:** Dimitrios Grivas, Álvaro González-Rajal, José Luis de la Pompa

**Affiliations:** ^1^Intercellular Signalling in Cardiovascular Development and Disease Laboratory, Centro Nacional de Investigaciones Cardiovasculares Carlos III (CNIC), Madrid, Spain; ^2^Ciber de Enfermedades Cardiovasculares, Madrid, Spain; ^3^Developmental Biology, Clinical, Experimental Surgery and Translational Research Center, Biomedical Research Foundation Academy of Athens, Athens, Greece; ^4^Genomics and Epigenetics Division, Garvan Institute of Medical Research, Sydney, NSW, Australia; ^5^St Vincent’s Clinical School, Faculty of Medicine, University of New South Wales, Sydney, NSW, Australia

**Keywords:** zebrafish heart regeneration, fibrotic scar, Midkine-a, signaling/signaling pathways, epicardium, collagen

## Abstract

Unlike the hearts of mammals, the adult zebrafish heart regenerates after injury. Heart cryoinjury in zebrafish triggers the formation of a fibrotic scar that gradually degrades, leading to regeneration. Midkine-a (Mdka) is a multifunctional cytokine that is activated after cardiac injury. Here, we investigated the role of *mdka* in zebrafish heart regeneration. We show that *mdka* expression was induced at 1-day post-cryoinjury (dpci) throughout the epicardial layer, whereas by 7 dpci expression had become restricted to the epicardial cells covering the injured area. To study the role of *mdka* in heart regeneration, we generated *mdka*-knock out (KO) zebrafish strains. Analysis of injured hearts showed that loss of *mdka* decreased endothelial cell proliferation and resulted in an arrest in heart regeneration characterized by retention of a collagenous scar. Transcriptional analysis revealed increases in collagen transcription and intense TGFβ signaling activity. These results reveal a critical role for *mdka* in fibrosis regulation during heart regeneration.

## Introduction

The adult mammalian heart has limited regeneration capacity, and myocardial infarction (MI) generates a permanent fibrotic scar that progressively leads to heart failure (Benjamin et al., 2019). Conversely, zebrafish can regenerate damaged cardiac tissue (Poss et al., 2002). Cryoinjury to the zebrafish ventricle results in massive cell death and the formation of scar tissue that gradually resolves, resulting in complete heart regeneration within 90 days (Schnabel et al., 2011; Chablais and Jazwinska, 2012b; Gonzalez-Rosa and Mercader, 2012). The most abundant extracellular matrix (ECM) component responsible for fibrotic tissue formation is collagen (Theocharis et al., 2016). Fibrosis progression is also dependent on the ECM molecule periostin, which promotes collagen cross-linking and fibroblast activation (Ito et al., 2014; Sanchez-Iranzo et al., 2018). In the injured zebrafish heart, the main sources of ECM are epicardial cells and epicardial-derived fibroblasts (Wang et al., 2013; Marro et al., 2016; Sanchez-Iranzo et al., 2018). TGFβ signaling, important for matricellular protein production, is active during regeneration, and chemical inhibition of the pathway decreases collagen deposition and blocks regeneration (Chablais and Jazwinska, 2012b). Heart regeneration is also prevented by inhibition of fibrotic tissue resolution (Xu et al., 2018), indicating that heart regeneration requires both the formation and the removal of the scar tissue.

*Midkine* (*mdk*) is a small, pleiotropic cytokine that is upregulated after injury in several tissues, including the heart, fin and retina (Lien et al., 2006; Luo et al., 2012). Cardiac insult in *Midkine-KO* mice leads to larger injuries and decreased heart function (Horiba et al., 2006; Ishiguro et al., 2011). Prolonged exposure of injured hearts to Midkine results in reduced collagen deposition in both the infarcted and healthy areas of the ventricle (Fukui et al., 2008; Takenaka et al., 2009; Sumida et al., 2010). Additionally, Midkine improves angiogenesis in injured cardiac tissue (Fukui et al., 2008; Takenaka et al., 2009; Sumida et al., 2010), indicating the importance of Midkine in the cardiac response to injury in mammals. Zebrafish have two Midkine genes, *midkine-a* (*mdka*) and its paralog *midkine-b* (*mdkb*), which during early development show complementary patterns of expression in the neural tube (Winkler et al., 2003). In the absence of *mdka*, retina regeneration is impaired, evidenced by decreased Muller glia proliferation and impaired photoreceptor regeneration (Nagashima et al., 2020). Fin regeneration is also delayed, due to an initial decrease in cell proliferation, although ultimately the fin is fully regenerated (Ang et al., 2020). Despite its importance during the cardiac response to injury in mammals, little is known about the role of *mdka* in zebrafish heart regeneration.

Here, we generated *mdka-KO* zebrafish and studied heart regeneration. We found that *mdka* is upregulated upon injury in the activated epicardium. Deletion of *mdka* decreased endothelial cell proliferation and prevented heart regeneration, leading to a persistent injured area enriched in collagen deposition. Transcriptional analysis revealed intense TGFβ signaling activity and increased expression of ECM molecules, including collagen and periostin, revealing a crucial role for *mdka* in the regulation of the fibrotic response upon heart injury.

## Results

### *mdka* Expression in the Regenerating Heart

**FIGURE 1:**
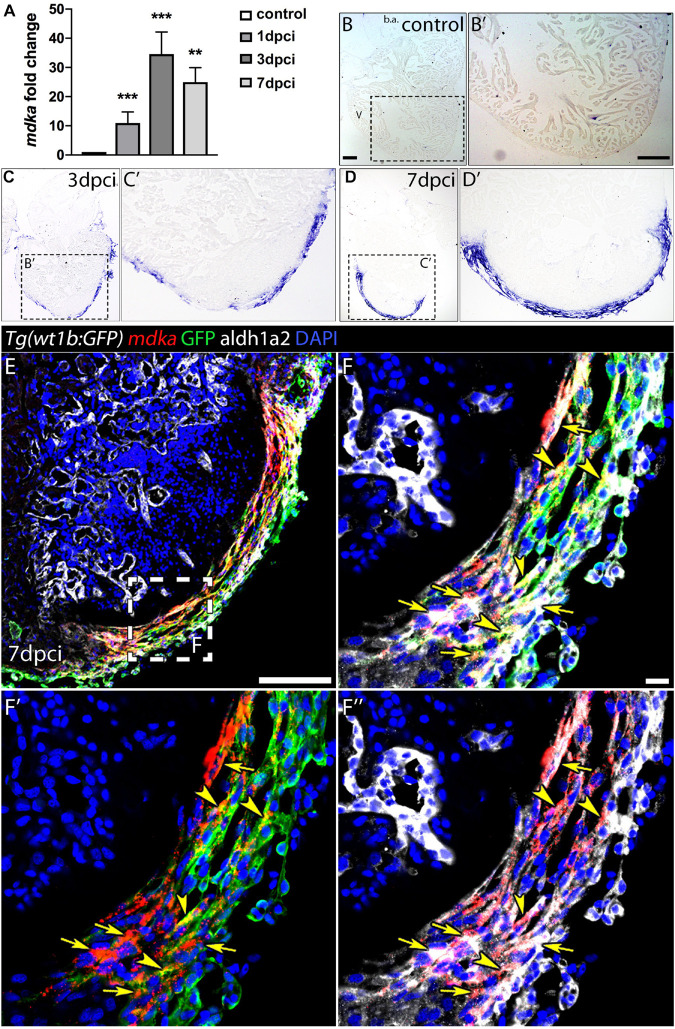
*mdka* expression upon injury. **(A)** qPCR analysis of *mdka* in regenerating hearts. Brown-Forsythe and Welch ANOVA tests; **P < 0.01, ***P < 0.001; Mean ± SD. **(B–D′)** ISH of *mdka* in intact **(B,B′)**, 3 dpci **(C,C′)**, and 7 dpci **(D,D′)** hearts. V, ventricle; b.a., bulbus arteriosus. Scale bars, 100 μm. **(E–F′′)** Fluorescent ISH for *mdka* followed by immunolabeling for GFP and Aldh1a2 in 7 dpci *Tg(wt1b:GFP)* hearts. Arrowheads indicate *mdka*-GFP overlap and arrows *mdka*-Aldh1a2 overlap. Scale bar E, 100 μm; **(F–F′′)**, 10 μm.

The expression of *mdka* has been reported to be upregulated upon heart injury in several animal models (Horiba et al., 2006; Lien et al., 2006; Fukui et al., 2008; Sumida et al., 2010). We therefore performed a quantitative PCR (qPCR) analysis in cryoinjured zebrafish hearts to analyse *mdka* expression during regeneration (Figure 1A). *mdka* RNA was already expressed in the heart at 1-day post-cryoinjury (dpci), reaching a peak at 3 dpci, followed by a slight reduction at 7 dpci. We next investigated the expression pattern of *mdka* by *in situ* hybridization (ISH) (Figures 1B–D′). At 1 dpci, *mdka* expression was detected in the epicardium, with no expression in intact hearts (Supplementary Figures 1A,A′ and Figures 1B,B′). At 3 dpci, *mdka* expression was activated in the epicardium, and by 7 dpci the expression became restricted to the multi-layered epicardium covering the injury site (Figures 1C–D′). Epicardial cells maintained *mdka* expression at 14 dpci (Supplementary Figures 1B,B′), and *mdka* remained detectable up to 130 dpci in the epicardium, the area between the cortical and trabecular myocardium, and in the compact layer, hinting at vascular expression (Supplementary Figures 1C,C′). To further define the epicardial localization of *mdka*, we performed fluorescence ISH in heart sections of *Tg(wt1b:GFP)* zebrafish, which express GFP in *wt1b*^+^ epicardial cells after injury (Gonzalez-Rosa et al., 2011). *mdka* ISH was followed by immunostaining for GFP and Aldh1a2, an enzyme involved in retinoid acid synthesis that is expressed in epicardial cells after injury (Kikuchi et al., 2011; Figures 1E–F′′). *mdka* was expressed in GFP^+^ cells (Figures 1F–F′′, arrowheads) and in Aldh1a2 expressing cells (Figures 1F′,F′′, arrows), indicating that injury triggers *mdka* expression in signaling cells. Our results thus show that *mdka* is highly upregulated in the epicardium upon injury and later persists in the activated epicardium.

### Generation of *mdka* Knock-Out Zebrafish

**FIGURE 2:**
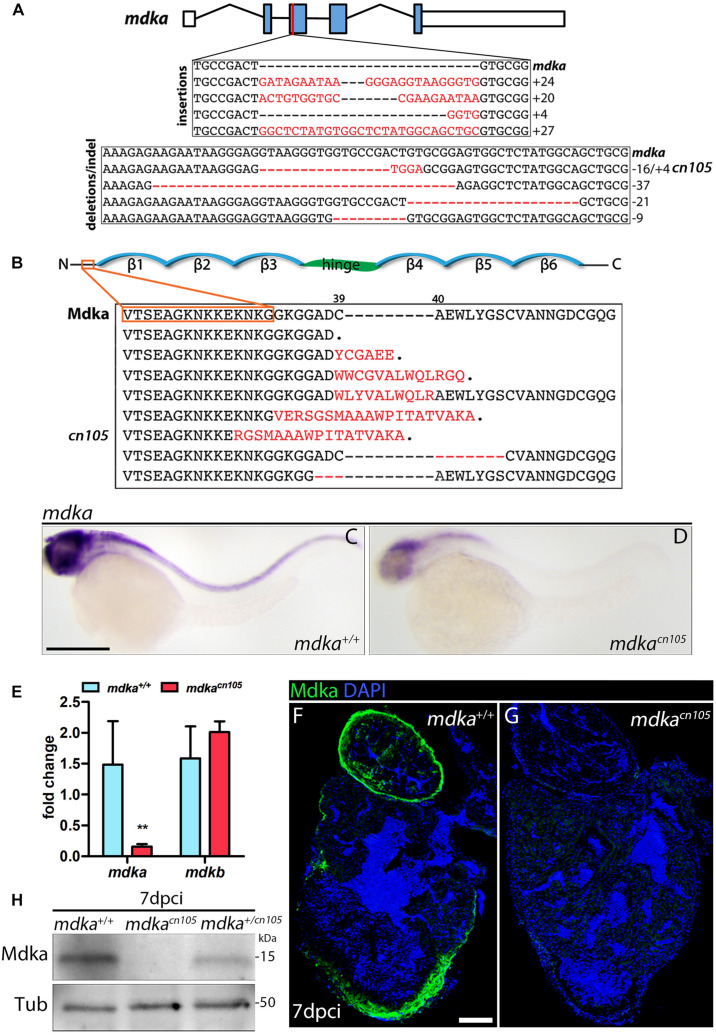
Generation of *mdka-KO* zebrafish. **(A)** The *mdka* locus and DNA mutations introduced by CRISPR/Cas9. The red line indicates the CRISPER target site, red letters indicate insertions, and red hyphens deletions. **(B)** Mdka domain organization and predicted protein mutations. Red letters denote novel amino acids, red hyphen deletions. β stands for beta-strands and the hinge domain is shown in green. **(C,D)** Whole-mount ISH (WM-ISH) for *mdka* in 2-day post-fertilization (dpf) *mdka*^+/+^ and *mdka*^*cn105*^ embryos. Scale bar, 200 μm. **(E)** qPCR analysis of *mdka* and *mdkb* in 2 dpf *mdka*^+/+^ and *mdka*^*cn105*^ embryos. *t*-test; **P < 0.01; Mean ± SD. **(F,G)** Immunofluorescence staining of Mdka in 7 dpci *mdka*^+/+^ and *mdka*^*cn105*^ hearts. Scale bar, 100 μm. **(H)** Western blot analysis for Mdka in 7 dpci *mdka*^+/+^, *mdka*^*cn105*^, and *mdka^+/cn105^* hearts. Tub, a-Tubulin; kDa, kilodalton.

In zebrafish, *mdka* is not expressed in the embryonic (Winkler et al., 2003) or adult heart; however, our initial analysis showed that *mdka* expression is readily activated in the epicardium after heart injury (Figure 1). The specific induction of high *mdka* expression upon injury suggested that loss of *mdka* might have a significant effect on heart regeneration. Therefore, we generated *mdka* knock-out (KO) zebrafish using CRISPR/Cas9 technology (Figure 2). The *mdka* gene is composed of five exons, and we targeted the third, which corresponds to the N-terminus of the protein (Figure 2A). Gene editing efficiency was high and resulted in equal numbers of deletion and insertion mutations (Figure 2A). Most of the predicted *mdka* mutations had a premature stop codon due to a frame shift in the open reading frame (Figure 2B). We selected the mutant allele *mdka*^*cn105*^ for further characterization (Figures 2A,B). This mutant allele results in severely decreased *mdka* expression, as revealed by whole-mount ISH (WM-ISH) and qPCR analysis (Figures 2C–E). Loss of *mdka* did not affect embryonic development, and *mdka*^*cn105*^ mutants were viable and fertile. We next used qPCR and WM-ISH to examine whether the absence of *mdka* triggered upregulation of *mdkb*. qPCR revealed no change in *mdkb* expression, and WM-ISH confirmed similar *mdkb* expression in *mdka*^+/+^ fish and *mdka*^*cn105*^ mutants (Figure 2E and Supplementary Figures 2A,B). To confirm that Mdka protein expression was lost, we conducted an immunofluorescence analysis of cryoinjured hearts from *mdka*^+/+^and *mdka*^*cn105*^ fish (Figures 2F,G). The strong epicardial expression of Mdka at 7 dpci in *mdka*^+/+^ hearts was completely absent in their *mdka*^*cn105*^ counterparts. Loss of Mdka was confirmed by western blot (WB) analysis (Figure 2H), which showed decreased post-injury Mdka expression in *mdka^+/cn105^* hearts and no expression in *mdka*^*cn105*^ hearts. Furthermore, expression of *mdkb* in *mdka*^*cn105*^ injured hearts was similar to that in *mdka*^+/+^ hearts (Supplementary Figures 2C,D), indicating that the *mdka*^*cn105*^ mutation abolishes *mdka* transcript and protein expression without upregulating potentially compensatory *mdkb* expression.

### *mdka-KO* Hearts Fail to Regenerate

**FIGURE 3:**
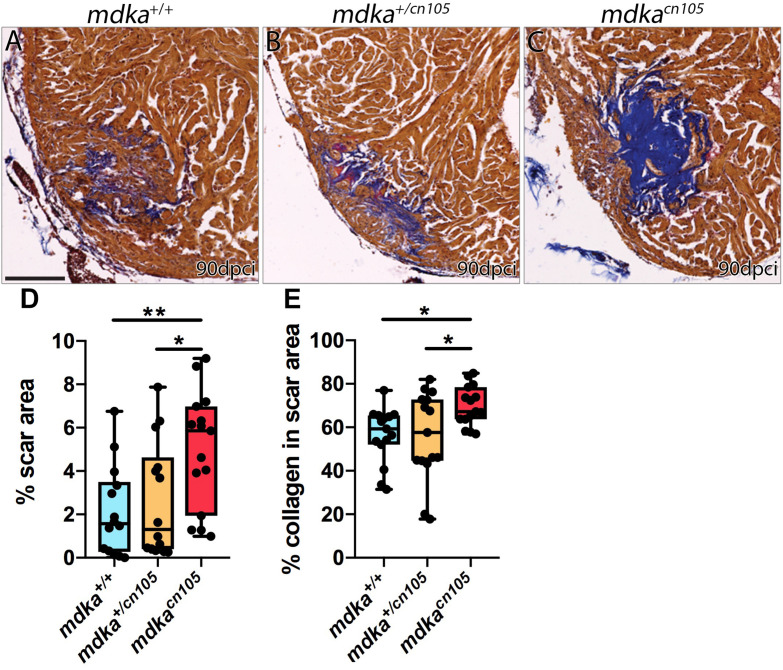
Loss of *mdka* leads to heart regeneration arrest. **(A–C)** AFOG staining of 90 dpci *mdka*^+/+^, *mdka^+/cn*105*^*, and *mdka*^*cn105*^ hearts. Collagen is shown in blue, fibrin in red, and healthy myocardium in brown. Scale bar, 100 μm. **(D)** Quantification of the scar area normalized to the total ventricle area. *n*_WT_ = 14, *n*_KO_ = 15, *n*_HET_ = 14. **(E)** Percentage of collagen in the scar area. *n*_WT_ = *n*_KO_ = *n*_HET_ = 15. Ordinary one-way anova; *P < 0.05; **P < 0.01; Mean ± SD.

We next examined the effect of *mdka* inactivation on heart regeneration. Hearts from *mdka*^+/+^, *mdka^*cn*10^*^5^, and *mdka^+/cn105^* animals were cryoinjured and allowed to regenerate for 90-days. Hearts were harvested and processed for Acid Fuchsin Orange-G (AFOG) staining, which labels collagen, fibrin, and healthy tissue (Figures 3A–C and Supplementary Figure 3). The analysis revealed that *mdka*^*cn105*^ hearts had significantly larger scar area than their *mdka*^+/+^ and *mdka^*cn105*/+^* counterparts (Figure 3D). Additionally, the scar area of *mdka*^*cn105*^ hearts was characterized by persistent collagen deposition compared with *mdka*^+/+^ and *mdka^*cn105*/+^* hearts (Figure 3E). These observations indicate that *mdka* deletion blocks heart regeneration. We further examined the regeneration of the caudal fin after amputation (Supplementary Figure 4). Loss of *mdka* led to a delay in fin regeneration, with fin outgrowth significantly smaller at 7 days post-amputation (dpa); however, regeneration was completed by 14 dpa (Supplementary Figure 4G), as reported previously (Ang et al., 2020).

### Increased Fibrosis in Injured *mdka*^*cn105*^ Hearts

**FIGURE 4:**
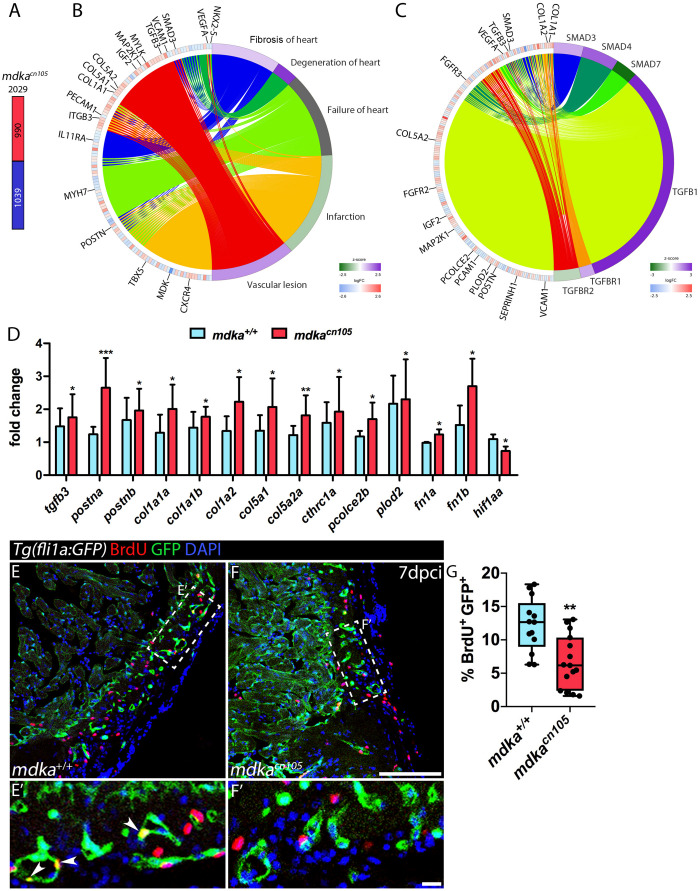
Transcriptional profiling of regenerating *mdka*^*cn105*^ hearts. **(A)** Total number of differentially expressed genes identified by RNA-seq. Numbers indicate upregulated genes (red) and downregulated genes (blue). **(B,C)** Circular plots showing representative differentially expressed genes (left semicircle perimeter) and IPA biofunctions and upstream regulators (right semicircle perimeter). Activation z-score scale: green, repression; magenta, activation; white, unchanged. LogFC scale: red, upregulated; blue, downregulated; white, unchanged. **(D)** qPCR analysis of *mdka*^+/+^ and *mdka*^*cn105*^ 7 dpci hearts. *t*-test; *P < 0.05, **P < 0.01, ***P < 0.001; Mean ± SD. **(E,F)** Immunofluorescence staining of GFP and BrdU in 7 dpci *Tg(fli1a:GFP)* heart sections. **(E′,F′)** Higher magnification of the dashed lines in **(A,F)**. Arrowheads indicate proliferating endothelial cells. Scale bar **(E,F)** 100 μm; **(E′,F′)** 10 μm. **(G)** Quantification of BrdU^+^/GFP^+^ endothelial cells. *n*_WT_ = 13, *n*_KO_ = 15; *t*-test; **P < 0.01; Mean ± SD.

To further study the effect of *mdka* deletion, we compared the transcriptional profile of 7 dpci *mdka*^+/+^ and *mdka*^*cn105*^ ventricles by RNA-seq. The analysis identified 2,029 differentially expressed genes, 990 of them upregulated and 1,039 downregulated (Figure 4A and Supplementary Table 1). Ingenuity pathway analysis (IPA) identified enrichment of processes involved in heart failure, including cardiac fibrosis, heart degeneration, and vascular lesions (Figure 4B). Analysis of upstream regulators suggested increased activation of the TGFβ pathway, including Tgfβ1, Tgfbr1, and Smad downstream effectors such as Smad3 and Smad4, whereas the TGFβ inhibitory effector Smad7 was reduced (Figure 4C; Moustakas and Heldin, 2009).

In *mdka*^*cn105*^ hearts, genes associated with fibrosis were significantly upregulated (Supplementary Figure 5). These included genes for the ECM components *col1a1a*, *col1a1b*, *col1a2*, *col5a1*, *col5a2a*, and *postna* (Chablais and Jazwinska, 2012b; Ito et al., 2014). Also upregulated were *pcolce2b*, *cthrc1a* and *plod2*, which are involved in collagen stability and scar tissue formation (Gilkes et al., 2013; Sorci-Thomas et al., 2015; Li et al., 2019). In contrast, expression of *vegfaa* and *hif1aa*, genes required for vascular development and repair, was decreased in *mdka*^*cn105*^ hearts (Marin-Juez et al., 2016; Gerri et al., 2017; Marin-Juez et al., 2019).

We validated our RNA-seq results by qPCR analysis using a different set of samples that also showed increased expression of *postnb*, *fibronectin1a* and *fibronectin1b* (Figure 4D). We confirmed the transcriptional upregulation of several collagens, including the fibrillar collagens *col1a1a*, *col1a1b*, *col1a2*, *col5a1*, and *col5a2a*, which are found in type I fibrils (Figure 4D; Theocharis et al., 2016). In addition, AFOG staining of 7 dpci hearts showed increased collagen deposition in *mdka*^*cn105*^ injured hearts (Supplementary Figure 6). The matricellular molecules *postna* and *postnb* were also upregulated in *mdka*^*cn105*^ hearts (Figure 4D). Periostin, which is expressed by cardiac fibroblasts is involved in formation of fibrillar collagen and contributes to fibroblast stimulation and persistency in injured hearts (Shimazaki et al., 2008; Sanchez-Iranzo et al., 2018; Dixon et al., 2019). The secretory function of activated fibroblasts, which secrete the ECM proteins that form fibrotic tissue, depends on TGFβ. Therefore, we evaluated TGFβ pathway activation by examining the downstream effector phospho-Smad3 (psmad3) (Supplementary Figure 7). Interestingly, the analysis revealed that phosphorylation of Smad3 was similar in *mdka*^+/+^ and *mdka*^*cn105*^ hearts, suggesting that *mdka* affects TGFβ activation through non-canonical pathways.

We next examined the proliferation of epicardial cells, because of their expression of *mdka*, and cardiomyocytes; however, neither cell type was significantly affected in *mdka* mutants (Supplementary Figure 7), suggesting that Mdka from epicardial or epicardial-derived cells is not required for the initial proliferation burst in the epicardium or in cardiomyocytes. Since Midkine is known to act as a pro-angiogenic factor (Sumi et al., 2002), we also analyzed the proliferation of cortical endothelial cells. We found that endothelial cells adjacent to the injury site proliferate less in *mdka*^*cn105*^ hearts than in control hearts (Figures 4E–G). In addition, we detected reduced transcriptional expression of *hif1aa* in *mdka*^*cn105*^ hearts (Figure 4D). HIF-1a has been shown to positively regulate *Midkine* expression and leads to enhanced angiogenesis (Reynolds et al., 2004), and zebrafish *hif1a* mutants have reduced endothelial cell proliferation in regenerating hearts (Marin-Juez et al., 2019). The decreased expression of *hif1aa* in *mdka*^*cn105*^ regenerating hearts can thus provide an explanation for the decline in endothelial cell proliferation.

Transcriptional analysis of injured *mdka*^*cn105*^ hearts revealed that loss of *mdka* led to upregulation of ECM molecules—including collagens, periostins and fibronectins—and intense TGFβ pathway activity. Additionally, the proliferation of the cortical endothelial cells was decreased, suggesting defective revascularization in the regenerating hearts. Therefore, increased fibrosis and persistent scar in *mdka*^*cn105*^ mutant hearts results from contributions of both increased ECM expression and angiogenesis defects.

## Discussion

Midkine is a highly conserved secreted factor that is induced upon injury of different tissues including muscle, retina, fin, and heart (Winkler et al., 2003; Lien et al., 2006; Luo et al., 2012; Ikutomo et al., 2014; Ang et al., 2020). Here, we generated *mdka-KO* zebrafish to study the role of Mdka in heart regeneration upon cryoinjury. Deletion of *mdka* resulted in arrest of heart regeneration and a scar area with increased collagen deposition. Analysis of *mdka*-deficient hearts showed transcriptional upregulation of ECM components such as collagens, periostins and fibronectins. Additionally, the proliferation of cortical endothelial cells was decreased, indicating defective angiogenesis.

Expression analysis revealed that *mdka* is not expressed in intact adult heart but it is highly upregulated upon injury. *mdka* expression was detected at 1 dpci throughout the epicardium, was maintained at 3 dpci, and by 7 dpci, the expression was restricted to epicardial cells surrounding the damaged tissue. Additionally, after the end of regeneration at 130 dpci, we also detected *mdka* in the newly formed compact layer, possibly in the vasculature, as well as in the zone between the cortical and trabecular myocardium, suggesting that regeneration requires prolonged *mdka* expression. This distinct spatio-temporal expression pattern contrasts with data from injured rodent hearts, which show *Mdk* expression in cardiomyocytes and/or endothelial cells, but not in epicardial cells (Obama et al., 1998; Fukui et al., 2008). In 7 dpci zebrafish hearts, the epicardium consists of a heterogeneous cell population that includes macrophages and fibroblasts (Sanchez-Iranzo et al., 2018; Sanz-Morejon et al., 2019). The main source of ECM is fibroblasts (Kendall and Feghali-Bostwick, 2014), and RNA-seq data from *postnb*^+^ cells show that *mdka* is highly upregulated in epicardial-derived fibroblasts in regenerating zebrafish hearts (Sanchez-Iranzo et al., 2018). *mdka* is also widely expressed in the epicardium, and epicardial cells secrete ECM components (Wang et al., 2013), suggesting that *mdka* is involved in scar tissue formation by epicardial and epicardial-derived cells.

To study the function of *mdka* in heart regeneration, we generated *mdka-KO* zebrafish using CRISPR/Cas9 genetic editing. *mdka*^*cn105*^ embryos developed and reached adulthood. Loss of *mdka* did not affect the transcription of the paralog *mdkb* in embryos. Mdka and Mdkb share 68% amino acid identity, and they show non-overlapping expression patterns in the embryos (Winkler et al., 2003). In the adult heart, we detected *mdka* expression only after cardiac injury, suggesting that *mdka* deletion will adversely affect heart regeneration. In *mdka*^*cn105*^ zebrafish, Mdka protein expression was lost in the injured heart without any accompanying activation of *mdkb*, suggesting that functional redundancy in the heart from *mdkb* is unlikely.

Cryoinjury of *mdka*^*cn105*^ hearts resulted in incomplete regeneration at 90 dpci, the final time-point of the regeneration process after cryoinjury. The persistent scar was characterized by the presence of collagen. Gene expression analysis showed that ECM molecules including *col1a1a*, *col1a1b*, *col1a2*, *col5a1*, *col5a2a*, *postna*, *postnb*, *fibronectin1a*, and *fibronectin1b* were upregulated. TGFβ induces transcription of collagens type I and V (Verrecchia et al., 2001). Upon heart cryoinjury in zebrafish, *tgf*β*1* is expressed in the injured area, and inhibition of TGFβ signaling leads to decreased collagen deposition and impairs regeneration (Chablais and Jazwinska, 2012b). Intriguingly, analysis of the TGFβ downstream effector Smad3 revealed comparable phosphorylation of Smad3 in *mdka*^*cn105*^ and *mdka*^+/+^ hearts, suggesting that the effect on the TGFβ pathway is via non-canonical pathways. Collagen is the main ECM scaffolding protein, and our analysis shows that collagen is upregulated in injured *mdka*^*cn105*^ hearts. Specifically, fibril-forming *col1a1a* and *col1a1b* encode pro-collagen chains that combine with the *col1a2* chain to produce type I procollagen molecules. Through enzymatic process, these procollagen molecules produce strong cross-linked fibers that contribute to scarring (Holmes et al., 2018), and these molecules are upregulated after *mdka* deletion. *mdka*^*cn105*^ hearts also showed upregulation of *postna* and *postnb*. In injured hearts, periostin is expressed by epicardial-derived fibroblasts and is involved in fibroblasts activation and persistence during regeneration (Sanchez-Iranzo et al., 2018; Dixon et al., 2019). Periostin is also involved in fibrosis through its stimulation of fibrillar collagen formation (Shimazaki et al., 2008). Also upregulated were *pcolce2b*, *cthrc1a*, and *plod2*. *pcolce2b* is involved in collagen-fibril assembly and ECM formation, is expressed in the epicardium after heart injury in zebrafish, and has been linked to Tgfβ1 stimulation and to pancreatic fibrotic disease (Cao et al., 2016). *cthrc1a* has been shown to contribute to fibrosis progression via TGFβ-dependent enhancement of liver fibrosis (Li et al., 2019), and *plod2* is involved in collagen fiber architecture and fibrosis development (Gilkes et al., 2013). These data indicate that *mdka* deletion triggers upregulation of genes responsible for scar formation and stability.

In zebrafish, heart regeneration after injury depends on both the formation and degradation of the scar tissue (Chablais and Jazwinska, 2012a; Sanchez-Iranzo et al., 2018; Xu et al., 2018). We found that *mdka* expression overlaps with the epicardial markers Wt1b and Aldh1a2, which are also co-expressed with *tcf21*; moreover, *tcf21*^+^ epicardial cells participate in collagen deposition (Kikuchi et al., 2011; Wang et al., 2013; Sanchez-Iranzo et al., 2018). Moreover, transcriptome analysis of *post1b*^+^ fibroblasts revealed that *mdka* is highly expressed by these cells (Sanchez-Iranzo et al., 2018). This suggests that heart regeneration arrest in *mdka-deficient* zebrafish is due to an increased fibrotic response, as evidenced by upregulation of collagens and periostin. This is consistent with findings in the injured mammalian heart, where treatment with Midkine after MI leads to decreased collagen deposition (Takenaka et al., 2009; Sumida et al., 2010). Additionally, *postnb*^+^ fibroblasts are detectable until 90 dpci, which could explain the persistent *mdka* expression that we observed after regeneration was complete. These data suggest that ECM regulation requires *mdka* expression by epicardial cells and epicardial-derived fibroblasts throughout the course of regeneration, and that *mdka* loss leads to increased ECM protein expression, resulting in impaired regeneration. An interesting idea is that *mdka* might be also involved in scar removal, and *mdka* inactivation result in a persistent collagen-rich scar. In this regard, treatment of human skin fibroblasts with Midkine results in increased *MMP2* transcription (Yamada et al., 1997). Future experiments will shed light on this issue.

We also found that *mdka*^*cn105*^ hearts have reduced transcriptional expression of the angiogenesis promoter *hif1aa*. Angiogenesis is necessary for revascularization, and defects in this process results in arrest of regeneration and maintenance of the fibrotic tissue (Harrison et al., 2015; Marin-Juez et al., 2016). *Midkine* contains HIF-response elements in its promoter and is positively regulated by HIF-1a to favor angiogenesis in embryonic mouse lungs (Reynolds et al., 2004). In addition, endothelial cell proliferation is reduced in *hif1a* zebrafish mutant hearts (Marin-Juez et al., 2019). These observations indicate that the reduced endothelial proliferation in *mdka*^*cn105*^ hearts is due to decreased *hif1a* expression. Fast revascularization is crucial for regeneration, since it provides the vascular network necessary for repopulation of the injured area, and coronary vasculature regeneration depends on epicardial signaling (Lavine et al., 2005; Kim et al., 2010). Hence, *mdka*, a secreted injury-induced cytokine, could act as a paracrine factor released from the epicardium and epicardial-derived fibroblast to induce revascularization of the damaged tissue.

Our analysis reveals that *mdka* is readily activated upon injury and is required for zebrafish heart regeneration. Thus, loss of *mdka* results in dysregulation of the fibrotic response and retention of the scar as well as defects in angiogenesis, leading to arrest of regeneration. These results suggest that *mdka* promotes cardiac remodeling through the regulation of collagen turnover and vascularization and show that regulated attenuation of ECM production is an essential step for fibrosis regression and complete regeneration.

## Materials and Methods

### Zebrafish Husbandry and Transgenic Lines

Animal studies were approved by the CNIC Animal Experimentation Ethics Committee and by the Community of Madrid (Ref. PROEX 83.8/20). Animal procedures conformed to EU Directive 2010/63EU and Recommendation 2007/526/EC regarding the protection of animals used for experimental and scientific purposes, enforced in Spanish law under Real Decreto 53/2013. Zebrafish were raised under standard conditions at 28°C (Kimmel et al., 1995). Fish lines used were the wild-type AB strain (ZIRC) and *Tg(wt1b:EGFP)^*li*1^* (Perner et al., 2007).

### Generation of *mdka* Mutant Alleles

*mdka-KO* zebrafish were generated using the oligonucleotides CACCATAGAGC CACTCCGCACAGT and AAACACTGTGC GGAGTGGCTCTAT, targeting the ACTGTGCGGAGTGG CTCTAT sequence. The oligos were inserted into the pX330 vector (Cong et al., 2013), which was linearized with *Bbs*I (New England Biolabs, Ipswich, MA). The guide mRNA was amplified with the forward primer ACGGGGTAATACGACTCACTATAGGGATAGAGCCACTCC GCACAG, which includes T7 polymerase promoter-specific sequences, and the reverse primer AAAAAGCACCGACTCGGT GCCA. The guide RNA was injected into one-cell stage embryos together with Cas9 protein (NEB). Mutants were identified by PCR using the following primers: *mdka*-Fwd TGTTATGTATGATTCTGCGAT and *mdka*-Rvs ACAGAGGCACAAAACTACCAA. The PCR product was examined in an agarose gel and the mutant animals were identified by the size difference of the PCR products. From the examined injected embryos, 91% were carrying deletions or insertions.

### Heart Cryoinjury and Fin Amputation

Fish were anaesthetized by immersion in fish water containing 0.04% tricaine (Sigma-Aldrich, St Louis, MO) and placed on a wet sponge under a dissecting microscope with the ventral side exposed. The cardiac cavity was dissected using microscissors and microforceps, and the pericardium was removed. The ventricle of the heart was exposed and dried and was then touched with a copper probe previously immersed in liquid nitrogen (Gonzalez-Rosa and Mercader, 2012). The fish were immediately returned to water to recover. Amputation of the caudal fin for regeneration experiments was performed as described (Munch et al., 2013). Briefly, fish were anaesthetized in 0.04% tricaine, and half of the caudal fin was amputated.

### Bromodeoxyuridine Injection

Adult fish were anaesthetized and placed on a wet sponge under a dissecting microscope. 5-bromo-2′-deoxyuridine (BrdU, Sigma-Aldrich, B5002) was diluted in phosphate-buffered saline (PBS) to 2.5 mg/ml, and 30 μl were injected intraperitoneally 24 h before heart dissection.

### Histology, ISH and Immunohistochemistry

Acid Fuchsin Orange G (AFOG) staining, *in situ* hybridization (ISH), fluorescent ISH, and whole-mount *in situ* hybridization (WM-ISH) were performed as described (Munch et al., 2017; Grivas et al., 2020). Primers for riboprobes used in this study are listed in Supplementary Table 2. For immunofluorescence, sections of paraffin-embedded tissue or cryosections were permeabilized with PBT (PBS containing 0.01% TritonX-100) and washed with PBS before incubation in blocking solution (2% bovine serum albumin, 10% goat serum, and 2 mM MgCl_2_ in PBS). Sections were then incubated overnight at 4°C with primary antibodies targeting BrdU (BD Transduction Laboratories, 347580, 1:30), GFP (Aves Labs, Tigard, GFP-1010, 1:200), Aldh1a2 (Gene Tex, GTX124302, 1:200), MEF2 (Santa Cruz Biotechnology, Santa Cruz, CA, sc-313, 1:100), phosho-Smad3 (Abcam, Cambridge, MA, ab52903, 1:200), or Mdka, 1:200 (Calinescu et al., 2009). Sections were then incubated with the appropriate secondary antibody and mounted after DAPI staining.

### Western Blot (WB)

Protein expression analysis by WB was performed as described (Grivas et al., 2020), using antibodies to Mdka (1:500) (Calinescu et al., 2009) and alpha-tubulin (Thermo Fisher Scientific, 62204, 1:5000).

### Image Analysis and Quantification

To analyze cardiomyocyte proliferation, MEF2^+^/BrdU^+^ nuclei were counted in a 100 μm area around the injury and normalized to the total MEF2^+^ cells. For the analysis of epicardial cell proliferation, epicardial/BrdU^+^ cells were counted and normalized to all the epicardial cells. For the coronary endothelial cell proliferation analysis, GFP^+^/BrdU^+^ cells were counted within a 200 μm radius of the injury site and normalized to the total GFP+ cells. For psmad3 quantification, psmad3+/epicardial cells were measured and normalized to the total epicardial cells. To quantify the scar area, the damaged (fibrotic tissue and collagen) and healthy (myocyte) areas were measured and normalized to the total ventricular area. The collagen—scar area index was calculated as the ratio of the collagen area to the whole scar area. At least three sections from the middle of the ventricle, containing at least on valve as anatomical references, of each heart were used for quantifications. For fin regeneration analysis, we measured the outgrowth of the regenerating fin from the amputation plane to the distal tip of the rays. All analyses were performed using Fiji (ImageJ, NIH).

### Gene Expression Analysis

Gene expression was analyzed by qPCR using the power SYBR Green Master Mix (Applied Biosystems, Foster City, CA) and the ABI PRISM 7900HT Real-Time PCR System. Each condition was analyzed using 3–4 biological replicates with 3 technical replicates per sample. Measurements were normalized to the expression of *18s* (in embryos) or of *elf1a* (in adult heart). All primers used for qPCR analysis are listed in Supplementary Table 3. RNA-seq analysis was conducted with 3 pools of 3 apexes from *mdka*^+/+^ or *mdka*^*cn105*^ hearts. cDNA libraries were prepared with the NEBNext Ultra II Directional RNA Library Prep Kit (New England Biolabs) and sequenced on a HiSeq 4000 system (Illumina) to generate 60 base single reads, and data were processed with RTA v1.18.66.3. FastQ files for each sample were obtained using bcl2fastq v2.20.0.422 software (Illumina). The resulting reads were mapped against the reference transcriptome GRCz11.99, and gene expression levels were estimated with RSEM (Li and Dewey, 2011). A single pairwise contrast was performed (KO vs. WT). To increase the number of potentially relevant genes, *p-*values were not corrected for multiple testing, and changes in gene expression were considered significant if associated with a raw *P-*value < 0.05. qPCR provided complementary evidence for the significant differential expression of selected genes, as described above. The resulting collection of 2,029 differentially expressed genes was used for functional enrichment analysis with IPA to derive overrepresented gene lists from Ingenuity’s proprietary knowledge-base (IPAKB). IPAKB-derived gene lists consist of collections of genes belonging to the same signaling or metabolic pathway (Canonical Pathway analyses) that either are regulated by the same molecule (Upstream Regulator analyses) or are associated with the same disease or biological function (Downstream Effect analysis). Enrichments associated with a Benjamini-Hochberg adjusted *P-*value < 0.05 were considered significant. Depending on the number or the type of genes involved, IPA also issued predictions about the activation state of pathways or regulators in the form of a parameter called the z-score; activation or inhibition is indicated by positive or negative values, respectively. Circular plots, summarizing the relationship between selected regulators and target differentially expressed genes were generated with the R package GOplot (Walter et al., 2015).

### Statistical Analysis

Sample sizes, statistical tests, and *P*-values are specified in the figure legends and were determined with GraphPad Prism software (GraphPad Software Inc., San Diego, CA). Statistical *t-*tests were two-tailed. Differences were considered statistically significant at *P* < 0.05.

## Data Availability Statement

Data are deposited in the NCBI GEO database under Accession Number GSE166732.

## Ethics Statement

The animal study was reviewed and approved by Animal studies were approved by the CNIC Animal Experimentation Ethics Committee and by the Community of Madrid (Ref. PROEX 83.8/20). Animal procedures conformed to EU Directive 2010/63EU and Recommendation 2007/526/EC regarding the protection of animals used for experimental and scientific purposes, enforced in Spanish law under Real Decreto 53/2013.

## Author Contributions

DG and ÁG-R performed experiments. DG and JLP designed the experiments, reviewed all the data, prepared the figures, and wrote the manuscript. All authors reviewed the manuscript during its preparation.

## Conflict of Interest

The authors declare that the research was conducted in the absence of any commercial or financial relationships that could be construed as a potential conflict of interest.

## Supplementary Material

Supplementary Figure 1 Expression of *mdka* in regenerating hearts. ISH of *mdka* in 1 dpci **(A,A′)**, 14 dpci **(B,B′)**, and 130 dpci **(C,C′)** hearts. V, ventricle; b.a., bulbus arteriosus. Scale bars, 100 μm.

Supplementary Figure 2 Analysis of *mdkb* in *mdka*^*cn105*^ embryos and injured hearts. **(A,B)** WM-ISH of *mdkb* in 2 dpf *mdka*^+/+^ or *mdka*^*cn105*^ embryos. **(C,D)** ISH of *mdkb* in 7 dpci *mdka*^+/+^ or *mdka*^*cn105*^ hearts. Scale bars **(A,B)** 200 μm; **(C,D)** 100 μm.

Supplementary Figure 3 *mdka*^*cn105*^ phenotype 90 dpci. AFOG staining of 90 dpci *mdka*^+/+^, *mdka^+/cn*105*^*, and *mdka*^*cn105*^ heart sections. Collagen in blue, fibrin in red, and healthy myocardium in brown. Section that contained at least one of the atrioventricular or the bulboventricular valves, were used for quantification of the scar area. Scale bar, 100 μm.

Supplementary Figure 4 Monitoring fin regeneration in *mdka*^*cn105*^ zebrafish. Fin regeneration at 3 dpa **(A,B)**, 7 dpa **(C,D)**, and 14 dpa **(E,F)** in *mdka*^+/+^ and *mdka*^*cn105*^ fish. Dashed lines indicate the amputation plane. Scale bars, 500 μm. **(G)** Quantification of fin outgrowth. 3 dpa, *n*_WT_ = 10, *n*_KO_ = 8; 7 dpa, *n*_WT_ = 5, *n*_KO_ = 6; 14 dpa, *n*_WT_ = 3, *n*_KO_ = 5; *t*-test; *^∗^P* < 0.05; Mean ± SD.

Supplementary Figure 5 RNA-seq analysis of 7 dpci hearts. RNA-seq log fold changes of ECM components and angiogenesis genes.

Supplementary Figure 6 Analysis of scar area and collagen in 7 dpci *mdka*^+/+^ and *mdka*^*cn105*^. **(A,B)** AFOG staining of 7 dpci *mdka*^+/+^ and *mdka*^*cn105*^ hearts. Collagen is shown in blue, fibrin in red, and healthy myocardium in brown. Scale bar, 100 μm. **(C)** Quantification of the scar area normalized to the total ventricle area. *n*_WT_ = *n*_KO_ = 4. **(D)** Percentage of collagen in the scar area. *n*_WT_ = 4, *n*_KO_ = 5; *t*-test; ^∗^*P* < 0.05; Mean ± SD.

Supplementary Figure 7 Phosphor-Smad3 quantification, epicardial cell and cardiomyocyte proliferation. **(A,B)** Immunofluorescence staining of *mdka*^+/+^ and *mdka*^*cn105*^ 7 dpci *Tg(wt1b:GFP)* heart sections for phosphor-Smad3 (psmad3, red) and GFP (green). **(C)** Quantification of psmad3^+^ epicardial cells (Epi). *n*_WT_ = 7, *n*_KO_ = 6; *t*-test; Mean ± SD. **(D,E)** Immunofluorescence staining of *mdka*^+/+^ and *mdka*^*cn105*^ 7 dpci *Tg(wt1b:GFP)* heart sections for BrdU (red), GFP (green), and Aldh1a2 (white). **(F)** Quantification of BrdU^+^ epicardial cells. *n*_WT_ = 10, *n*_KO_ = 11; *t*-test; Mean ± SD. **(G,H)** Immunofluorescence staining of BrdU and MEF2 in 7 dpci *mdka*^+/+^ and *mdka*^*cn105*^ hearts. Dashed lines indicate the quantification area. **(I)** Quantification of BrdU^+^/MEF2^+^ cardiomyocytes (CM). *n*_WT_ = 6, *n*_KO_ = 7; *t*-test; Mean ± SD; Scale bars, 100 μm.
