# Supplementary figures and images for: Midkine-a Regulates the Formation of a Fibrotic Scar During Zebrafish Heart Regeneration
Source: Front Cell Dev Biol. 2021 May 7;9:669439. doi: 10.3389/fcell.2021.669439 (PMC8138450; doi:10.3389/fcell.2021.669439)

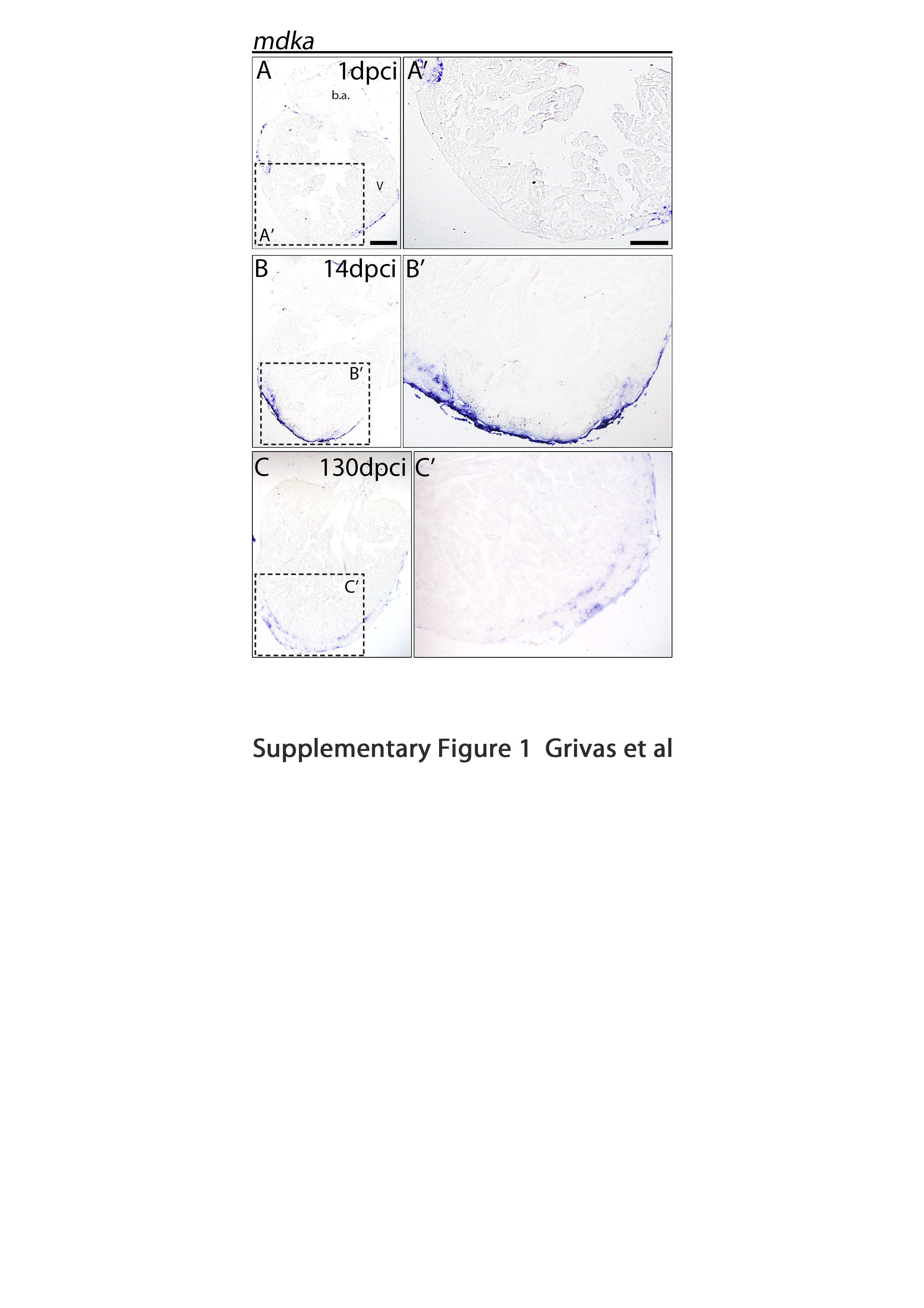

Supplement: Supplementary Figure 1 — Expression of mdka in regenerating hearts. ISH of mdka in 1 dpci (A,A′), 14 dpci (B,B′), and 130 dpci (C,C′) hearts. V, ventricle; b.a., bulbus arteriosus. Scale bars, 100 μm. [file Image_1.JPEG]

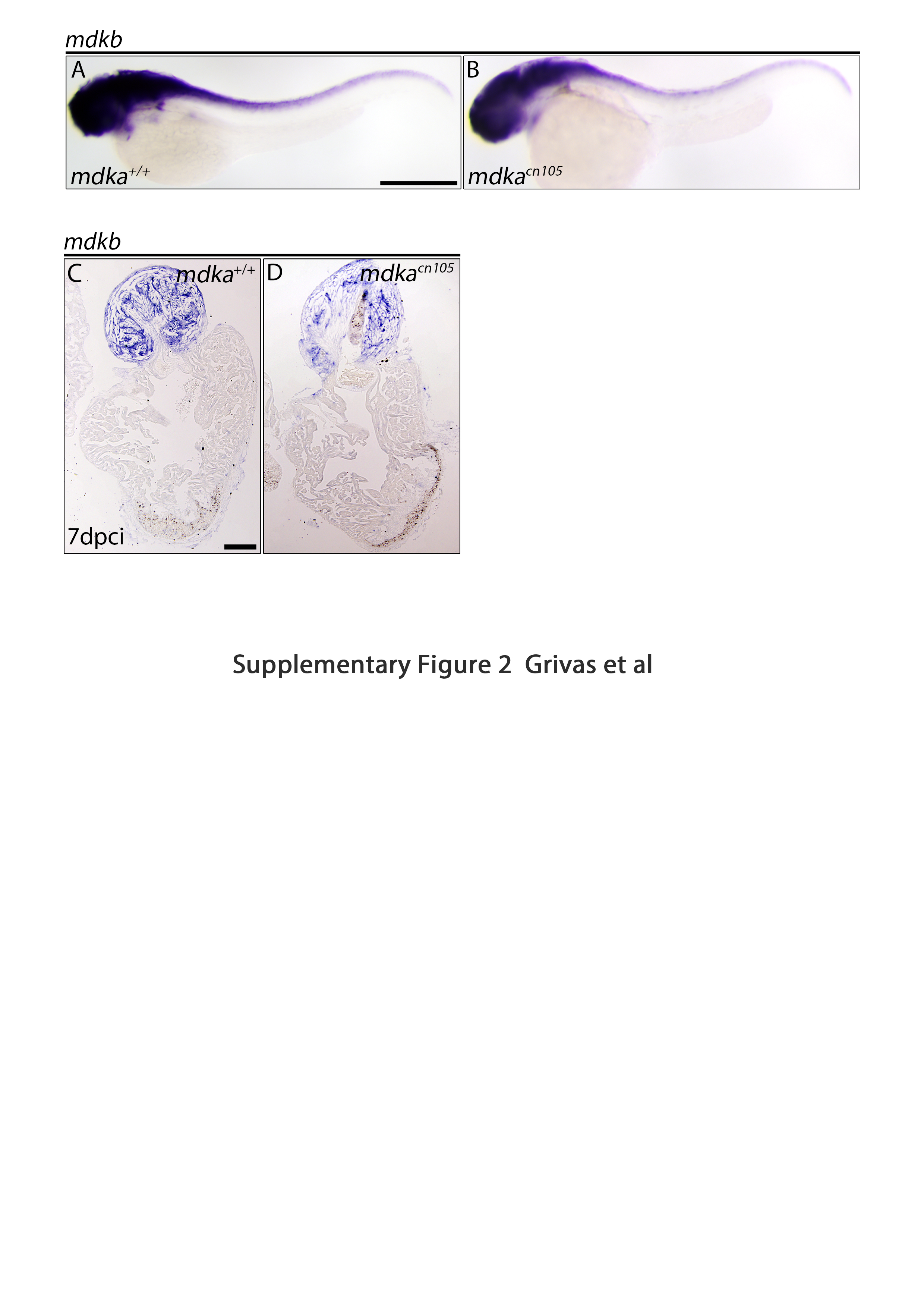

Supplement: Supplementary Figure 2 — Analysis of mdkb in mdkacn105 embryos and injured hearts. (A,B) WM-ISH of mdkb in 2 dpf mdka+/+ or mdkacn105 embryos. (C,D) ISH of mdkb in 7 dpci mdka+/+ or mdkacn105 hearts. Scale bars (A,B) 200 μm; (C,D) 100 μm. [file Image_2.JPEG]

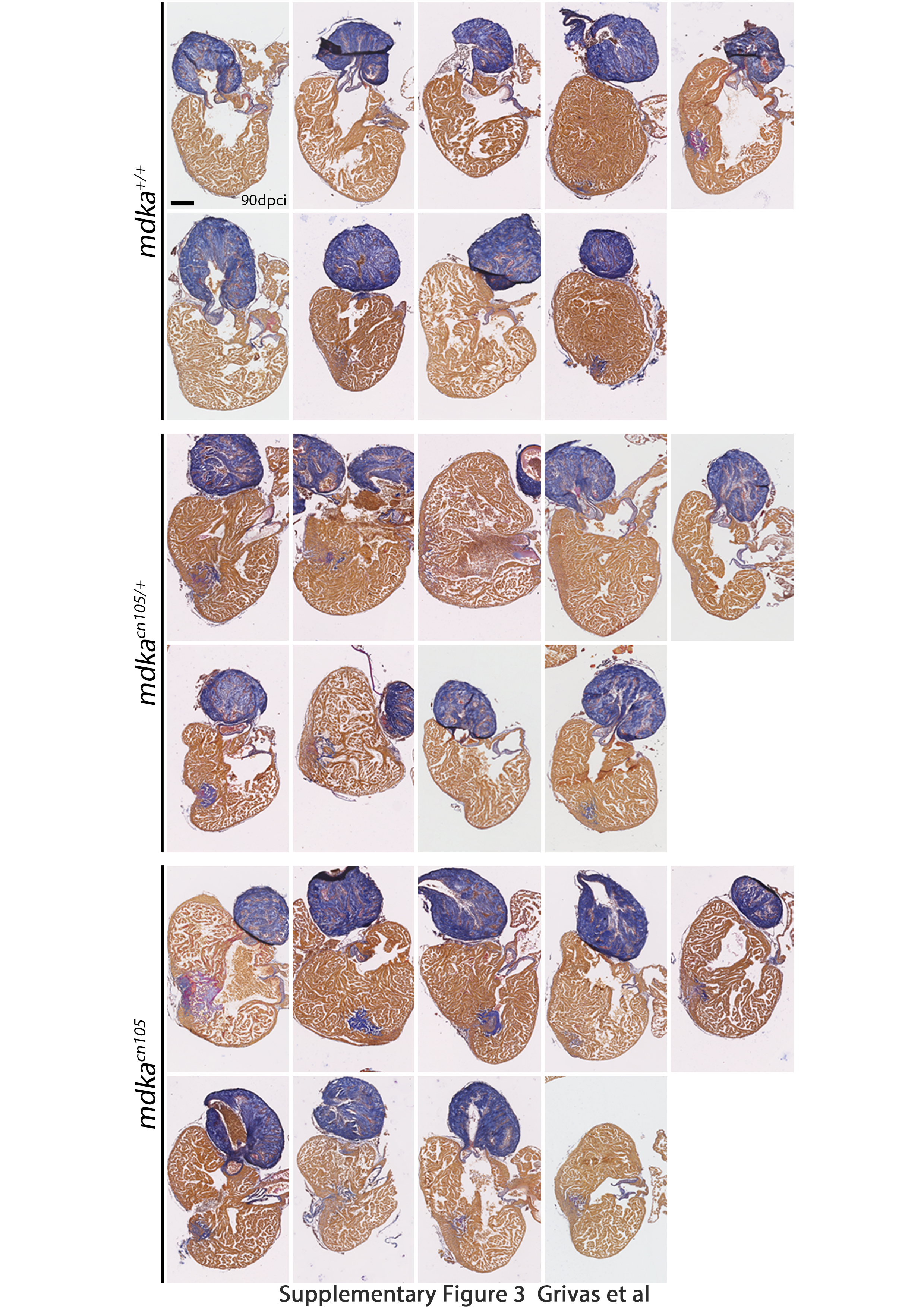

Supplement: Supplementary Figure 3 — mdkacn105 phenotype 90 dpci. AFOG staining of 90 dpci mdka+/+, mdka+/cn105, and mdkacn105 heart sections. Collagen in blue, fibrin in red, and healthy myocardium in brown. Section that contained at least one of the atrioventricular or the bulboventricular valves, were used for quantification of the scar area. Scale bar, 100 μm. [file Image_3.JPEG]

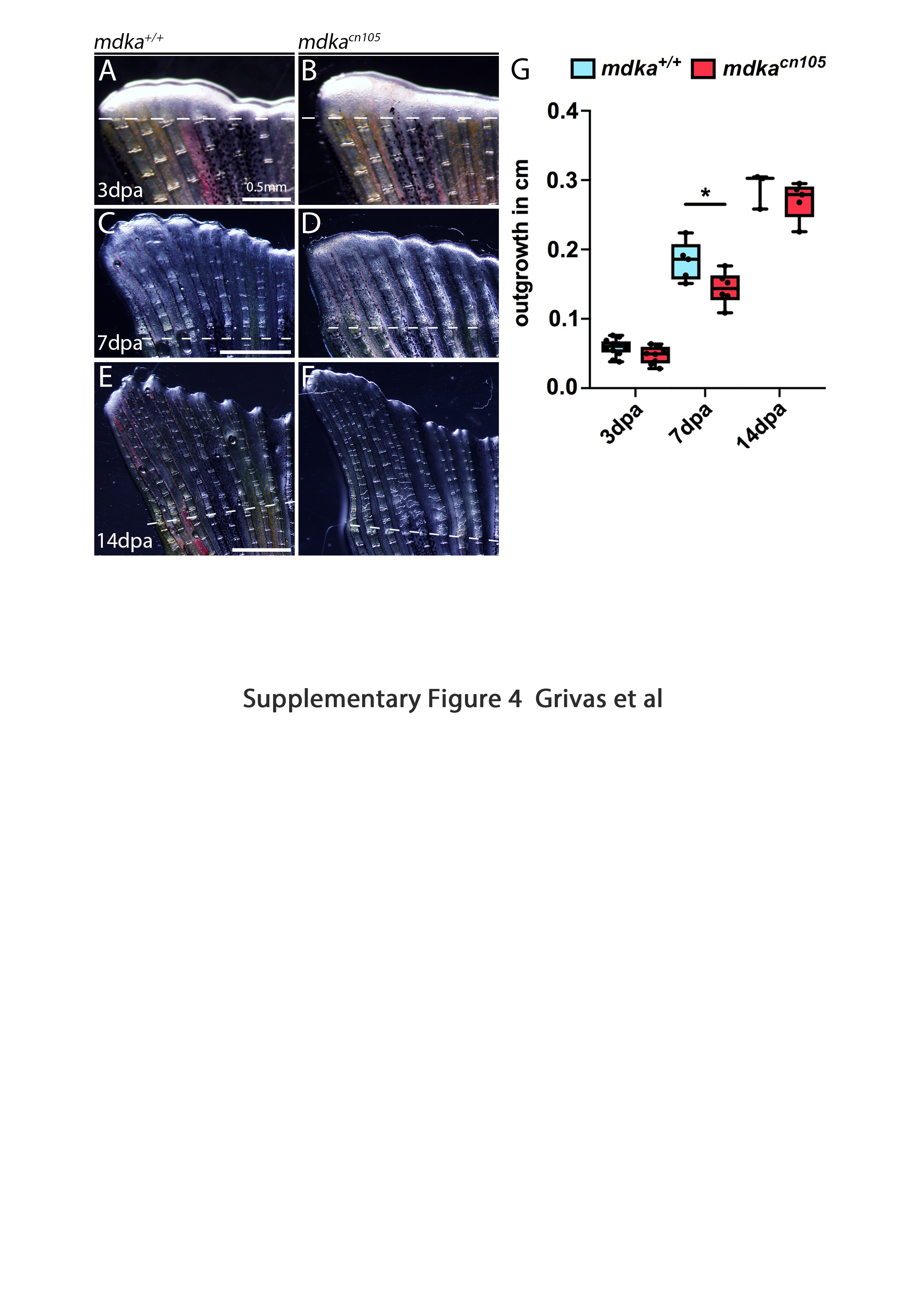

Supplement: Supplementary Figure 4 — Monitoring fin regeneration in mdkacn105 zebrafish. Fin regeneration at 3 dpa (A,B), 7 dpa (C,D), and 14 dpa (E,F) in mdka+/+ and mdkacn105 fish. Dashed lines indicate the amputation plane. Scale bars, 500 μm. (G) Quantification of fin outgrowth. 3 dpa, nWT = 10, nKO = 8; 7 dpa, nWT = 5, nKO = 6; 14 dpa, nWT = 3, nKO = 5; t-test; ∗P < 0.05; Mean ± SD. [file Image_4.JPEG]

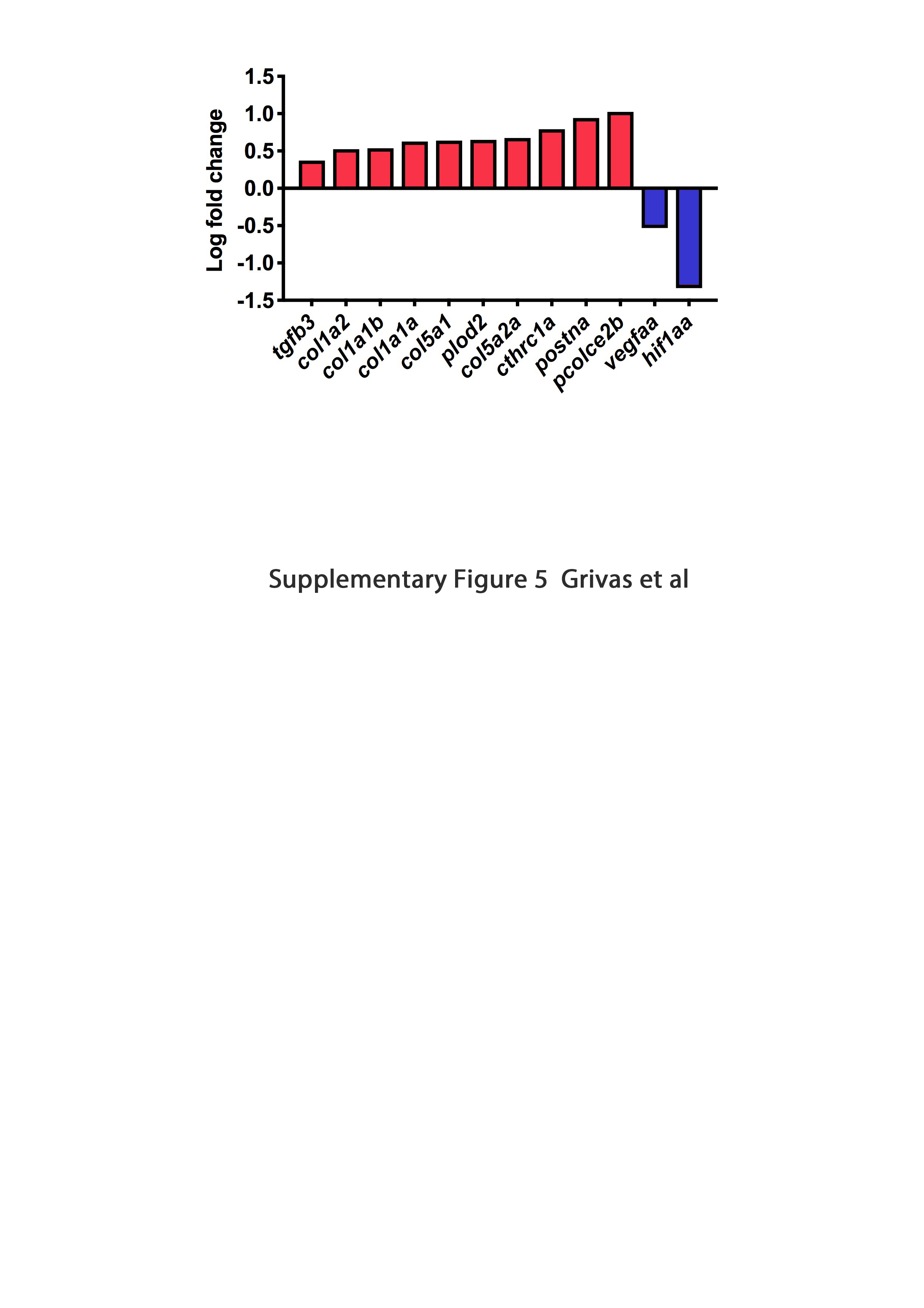

Supplement: Supplementary Figure 5 — RNA-seq analysis of 7 dpci hearts. RNA-seq log fold changes of ECM components and angiogenesis genes. [file Image_5.JPEG]

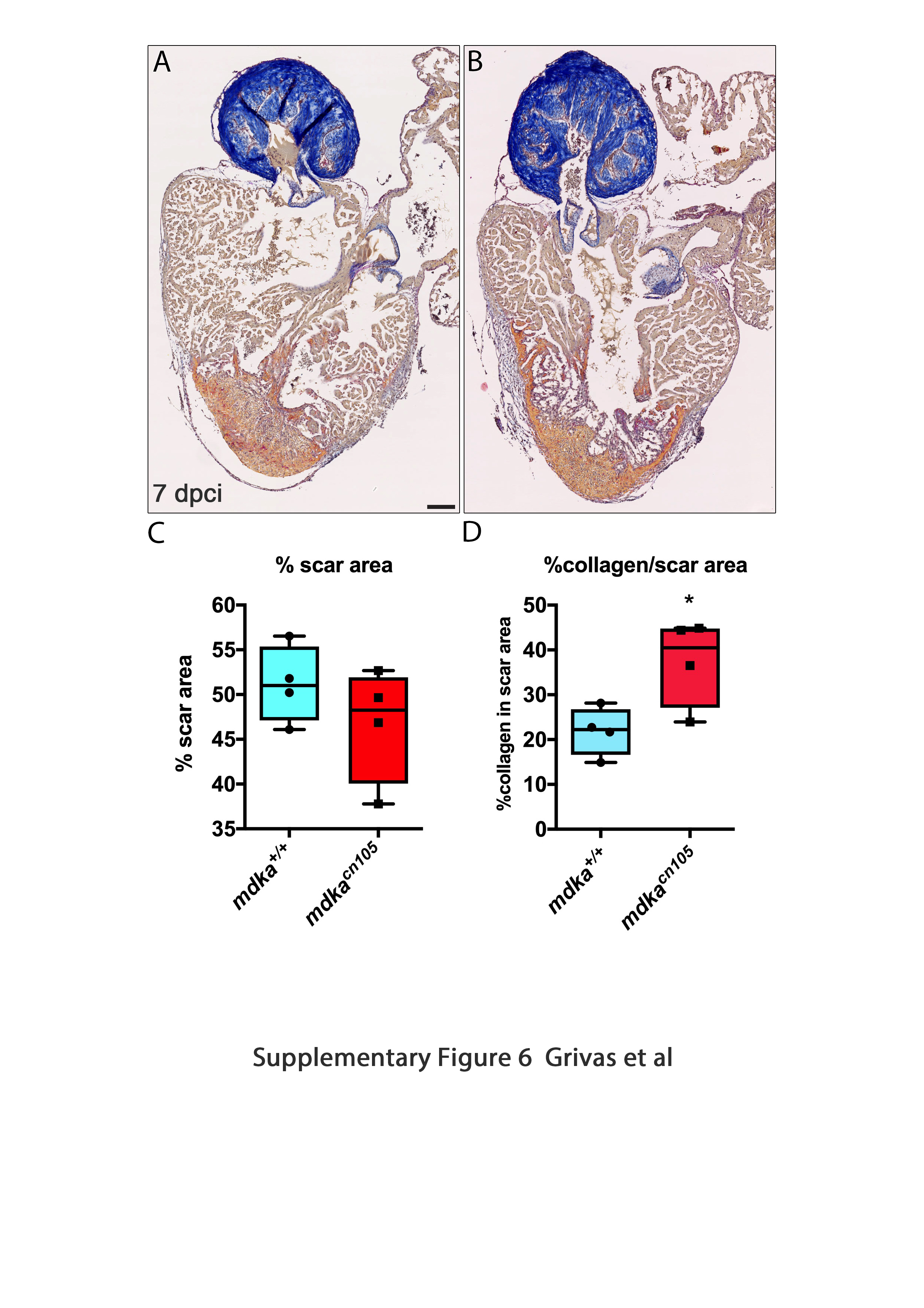

Supplement: Supplementary Figure 6 — Analysis of scar area and collagen in 7 dpci mdka+/+ and mdkacn105. (A,B) AFOG staining of 7 dpci mdka+/+ and mdkacn105 hearts. Collagen is shown in blue, fibrin in red, and healthy myocardium in brown. Scale bar, 100 μm. (C) Quantification of the scar area normalized to the total ventricle area. nWT = nKO = 4. (D) Percentage of collagen in the scar area. nWT = 4, nKO = 5; t-test; ∗P < 0.05; Mean ± SD. [file Image_6.JPEG]

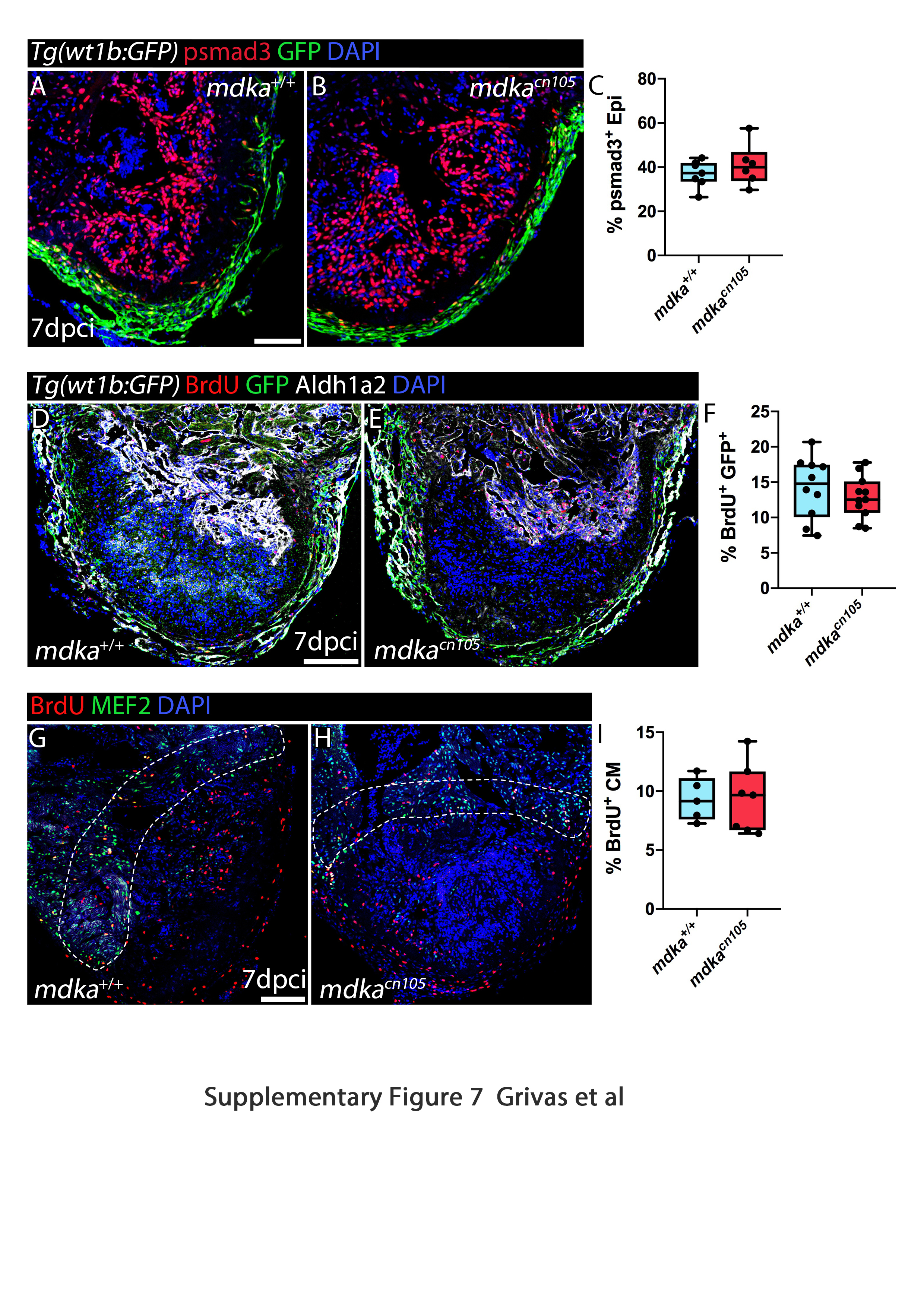

Supplement: Supplementary Figure 7 — Phosphor-Smad3 quantification, epicardial cell and cardiomyocyte proliferation. (A,B) Immunofluorescence staining of mdka+/+ and mdkacn105 7 dpci Tg(wt1b:GFP) heart sections for phosphor-Smad3 (psmad3, red) and GFP (green). (C) Quantification of psmad3+ epicardial cells (Epi). nWT = 7, nKO = 6; t-test; Mean ± SD. (D,E) Immunofluorescence staining of mdka+/+ and mdkacn105 7 dpci Tg(wt1b:GFP) heart sections for BrdU (red), GFP (green), and Aldh1a2 (white). (F) Quantification of BrdU+ epicardial cells. nWT = 10, nKO = 11; t-test; Mean ± SD. (G,H) Immunofluorescence staining of BrdU and MEF2 in 7 dpci mdka+/+ and mdkacn105 hearts. Dashed lines indicate the quantification area. (I) Quantification of BrdU+/MEF2+ cardiomyocytes (CM). nWT = 6, nKO = 7; t-test; Mean ± SD; Scale bars, 100 μm. [file Image_7.JPEG]
