# Supplementary material for: Midkine-a Regulates the Formation of a Fibrotic Scar During Zebrafish Heart Regeneration
Source: Front Cell Dev Biol. 2021 May 7;9:669439. doi: 10.3389/fcell.2021.669439 (PMC8138450; doi:10.3389/fcell.2021.669439)
Supplement: Supplementary file 9 [file Table_2.docx]

**Supplementary Table 2. Primers for riboprobes.**

| **gene** | **Forward** | **Reverse** | **Reference** |
| --- | --- | --- | --- |
| mdka | CAAAGGAAAGAAAGGCAAGGGG  AAAGGGAACTAAAGG | GACTTTCCAATTGCTACTTTTATACCCC  GCTAGTAAT | This paper. |
| mdkb | CCCAATAGCGGAGACTGT | ATCTTGCCAGTTTTCCTCT |  |
