# Supplementary material for: Midkine-a Regulates the Formation of a Fibrotic Scar During Zebrafish Heart Regeneration
Source: Front Cell Dev Biol. 2021 May 7;9:669439. doi: 10.3389/fcell.2021.669439 (PMC8138450; doi:10.3389/fcell.2021.669439)
Supplement: Supplementary file 10 [file Table_3.docx]

**Supplementary Table 3. qPCR primers**

| **gene** | **Forward** | **Reverse** | **Reference** |
| --- | --- | --- | --- |
| mdka | ACCGTCTCTGTGACCAAACC | CTTTCCCCTTGCCTTTCTTT | This report |
| mdkb | CAGCATCATATTCACAGCAA | AGAGCTATAGAGAACAAACTCC |  |
| col5a2a | TCAGGAACCTCTGGCCCTAA | TCCAGGCGTACCTGTCAAAC |  |
| cthrc1a | CACTGGCCCGTTACCCATAG | CCACTGTGGATGTTCTGTGGA |  |
| pcolce2b | CACCAGACCCACACCTTTGA | AGTAGTGGCTCTCAGGGGTT |  |
| plod2 | TAGATTTCCTGGGCGGAGGT | GAGTGAGTCGACCTGGATGC |  |
| postnb | TCCAGGGAGAGCCTACCATC | GCTGACCCTCAATCACACGA |  |
| col1a2 | AAGAACCCCGCTCGTACTTG | TCCAGTAGAAACCGCTGCTC |  |
| tgfb3 | AATGGCTGCAGGGTTCA | GGTTTGCTTTACAGTCGCAGT |  |
| postna | GGGAAACAACCTTCAAGTGCT | TGGACTACTCCATTGGATGCC | (Narumanchi et al., 2019) |
| col1a1a | GCTTTTGGCAAGAGGACAAG | TGTCTTCGCAGATCACTTCG | (Landgraf et al., 2017) |
| col1a1b | CCTGGTGCTGCTGGTATTGC | TCTCCATTGTTTCCTTTGGGG | (Zhang et al., 2016) |
| col5a1 | CACCCTATGCCTTATCAGTCTTC | TGTTTCATTTGCTCAATCTCCA | (Hoffman et al., 2010) |
| fn1a | GGAGAACGCTCTACGCAAAC | AGGTCTGGTCTCAGGCACTC | (Burczyk et al., 2015) |
| fn1b | CAGCAGTCCCCCTCTTACTG | CATGACACCTGGATGCAAAC | (Danilova et al., 2018) |
| vegfaa | CGAGAGCTGCTGGTAGACATC | GGATGTACGTGTGCTCGATCT | (El-Brolosy et al., 2019) |
| hif1aa | AGCCGCCACACTTTAGACAT | CCTCTGGATCAAAACCCAAG | (Gerri et al., 2017) |
